# Precise pigment biosynthesis for flower color design in *Brassica napus*

**DOI:** 10.1093/hr/uhaf193

**Published:** 2025-07-29

**Authors:** Yuhan Zheng, Rui Shi, Wan Chen, Xinfa Wang, Xiaoling Dun, Hanzhong Wang, Jinwu Deng

**Affiliations:** Key Laboratory of Biology and Genetic Improvement of Oil Crops, Oil Crops Research Institute of the Chinese Academy of Agricultural Sciences, Ministry of Agriculture and Rural Affairs, Wuhan 430062, China; Key Laboratory of Biology and Genetic Improvement of Oil Crops, Oil Crops Research Institute of the Chinese Academy of Agricultural Sciences, Ministry of Agriculture and Rural Affairs, Wuhan 430062, China; Key Laboratory of Biology and Genetic Improvement of Oil Crops, Oil Crops Research Institute of the Chinese Academy of Agricultural Sciences, Ministry of Agriculture and Rural Affairs, Wuhan 430062, China; Key Laboratory of Biology and Genetic Improvement of Oil Crops, Oil Crops Research Institute of the Chinese Academy of Agricultural Sciences, Ministry of Agriculture and Rural Affairs, Wuhan 430062, China; Key Laboratory of Biology and Genetic Improvement of Oil Crops, Oil Crops Research Institute of the Chinese Academy of Agricultural Sciences, Ministry of Agriculture and Rural Affairs, Wuhan 430062, China; Key Laboratory of Biology and Genetic Improvement of Oil Crops, Oil Crops Research Institute of the Chinese Academy of Agricultural Sciences, Ministry of Agriculture and Rural Affairs, Wuhan 430062, China; Hubei Hongshan Laboratory, Wuhan 430070, China; Key Laboratory of Biology and Genetic Improvement of Oil Crops, Oil Crops Research Institute of the Chinese Academy of Agricultural Sciences, Ministry of Agriculture and Rural Affairs, Wuhan 430062, China

## Abstract

The flower color has drawn extensive attention in rapeseed breeding for its ornamental value. However, the color formation and precise design are still elusive. Here, we successfully introduced betalain biosynthesis pathway into rapeseed and achieved constitutive betalain production by overexpressing *RUBY*. The varying expression levels of *RUBY* and the flower colors of the receptor materials jointly determined the final color presentation. When *RUBY* was expressed in yellow-flowered rapeseed (cultivar R10), the flower color turned into different shades of orange. In white-flowered background (cultivar R2), *RUBY* created red flowers. However, *RUBY* overexpression led to dark-red leaves and decreased photosynthesis. To recover normal photosynthesis, we created orange flowers with green leaves using petal-specific *XY355* promoter in yellow-flowered R10. We further verified that white flower is dominant to yellow and created green leaves with shining red flowers by crossing orange flower (*XY355:RUBY* expressed in yellow background) with white flower (R2). Given that the widespread carotenoid, betalain, and anthocyanin can produce the three major colors of yellow, red, and blue, respectively, we provide a promising approach for creating derivative colors in *Brassica napus* by employing bioengineering approaches to precisely regulate the pigment biosynthesis.

## Introduction

The economic importance of ornamental plants is increasing worldwide. According to the International Statistics Flowers and Plants yearbook, the ornamental horticulture industry was valued at $70 billion in 2024 [1]. Rapeseed (*Brassica napus*) is the third largest oil crop around the world. In addition to traditional oil purpose, the rise of sightseeing tourism leads to an increasingly urgent demand for breeding colorful flower varieties. Traditionally, the flower color of cultivated rapeseed mainly ranges from white to different shades of yellow, according to the content of carotenoid [2]. Recently, new rapeseed flower colors such as orange, apricot, pink, and purple have been developed through distant hybridization with other species like *Raphanus sativus* or *Orychophragmus violaceus*. However, their flower color generated through this method are usually faded and accompanied by high contents of erucic and glucosinolates in seeds, which are not suitable for edible oil or forage [3, 4]. Though other approaches including EMS mutagenesis and genetic engineering are successfully used in flower color improvement for horticultural plants, there are only a few cases in rapeseed and the changes of rapeseed flower color are subtle [5, 6].

Carotenoid, betalain, and anthocyanin are known as the three major pigments in plants [7]. Carotenoids are a class of fat-soluble pigments that are widely distributed in nature. More than 700 different types of carotenoids have been identified [8], ranging from colorless to yellow, orange, and red. Notable examples include lycopene in tomato, β-carotene in carrot, zeaxanthin in corn, and lutein in marigold [7, 8]. The functional deficiency of an R2R3-MYB protein (WP1) impairs carotenoid biosynthesis in *Medicago truncatula*, causing the petals to change from yellow to white [9]. CsMADS5 regulates carotenoid biosynthesis during citrus fruit ripening, thereby controlling fruit coloration [10]. In rapeseed, the concentration of carotenoid in yellow petals is generally higher than that in white. BnaPDS3 and BnaCRTISO are involved in carotenoid biosynthesis, and their mutations cause yellow-to-white petal color changes due to decreased carotenoid levels [5, 11]. However, BnaC3.CCD4 is suggested to play a key role in carotenoid degradation by cleaving α- and/or δ-carotenes into volatile α-ionone in petals. Its mutation results in the transformation of petal color from white to yellow [12]. In addition, *BnNCED4b* is also involved in carotenoid degradation, and its expression leads to decreased levels of lutein and zeaxanthin, thus making flowers turn white [13].

Betalains are a class of water-soluble pigments, typically divided into betacyanins and betaxanthins, characterized by red-violet and yellow-orange colors, respectively. Betalain is mainly found in the order of *Caryophyllales*, including beets, dragon fruit, and cactus fruit [14]. Betalain can be synthesized from tyrosine by three enzymes (CYP76AD1, L-DOPA 4,5-dioxygenase, glucosyltransferase) [15]. During pulp maturation, the above-mentioned genes were specifically highly expressed in betalain-rich ‘GHH’ pitaya (red peel and red pulp) compared to ‘GHB’ pitaya (red peel and white pulp) [16]. A novel reporter system named *RUBY*, which carries these three genes in a single open reading frame, was successfully developed and introduced into Arabidopsis, rice, and tobacco [17]. Moreover, it was also effectively expressed in silkworm (*Bombyx mori*), displaying a robust biocompatibility [18].

Anthocyanin, a subclass of flavonoids, contributes to approximately 88% of flower color variation, including orange, red, purple, and blue [19]. Chalcone synthase (CHS), chalcone isomerase (CHI), and flavanone 3-hydroxylase (F3H) are crucial for the production of flavonoid precursors. Flavonoid 3′-hydroxylase (F3′H), flavonoid 3′5′-hydroxylase (F3′5′H), dihydroflavonol 4-reductase (DFR), anthocyanidin synthase (ANS), and flavonoid 3-O-glucosyltransferase (UFGT) are responsible for the biosynthesis of specific anthocyanin classes [20], including malvidin, delphinidin, petunidin, peonidin, cyanidin, and pelargonidin. Among these pigments, delphinidin is highly related to blue flowers [7, 21]. It is worth mentioning that the gene *F3*′*5*′*H* is crucial for delphinidin synthesis and heterologous expression of *F3*′*5*′*H* (from blue/violet flowered *Petunia*, *Viola*, or *Campanula*) in carnations, roses, and chrysanthemums successfully created new blue-flower cultivars [22–24]. Previous studies on rapeseed with colorful flowers have demonstrated that anthocyanins are synthesized in petal tissues. Specifically, F3′H directly participates in anthocyanin synthesis, promoting anthocyanin accumulation in purple-flowered rapeseed [25]. Meanwhile, BnaA07.PAP2 activates genes involved in anthocyanin synthesis in apricot and pink rapeseed varieties [26]. In addition, heterologous expression *OvPAP2* (*O. violaceus*) in *B. napus* can also promote anthocyanin accumulation in petals [6].

Growing evidence suggests that it is possible to manipulate pigment metabolism in flowers. In this study, we introduced the betalain biosynthetic gene cluster *RUBY* into rapeseed and achieved constitutive or specific expression using different promoters. In particular, petal-specific promoter *XY355* enabled the precise biosynthesis of betalain in flower petals, which avoided its interference with photosynthesis in leaves. We also demonstrated that betalain can be used to creat derivative colors together with the background color. More specifically, when *RUBY* was expressed in a white-flowered background, we obtained red flowers. When *RUBY* was introduced into yellow-flowered background, the orange flower was created. Here, we have successfully obtained white, yellow, red, and their combination of orange flowers. In addition, it is possible to create blue delphinidin by changing anthocyanin metabolic pathways under existed anthocyanin abundant flowers. By leveraging the independent metabolic pathways of three major pigments: carotenoid (yellow), betalain (red), and anthocyanin (blue), we propose a strategy for designing derivative flower colors in *B. napus* and potentially other horticultural plants.

## Results

### Betalain biosynthesis in *B. napus*

**Figure 1:**
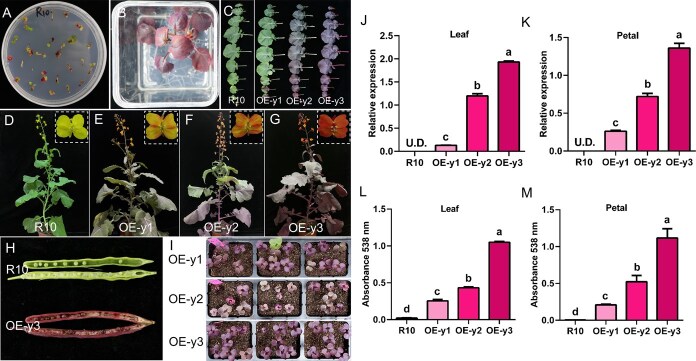
*35S:RUBY* expressed in R10 background. (A) *RUBY* was expressed in R10 callus. (B) *Ruby* transgenic seedlings were obtained on the rooting medium. (C) The seedling stage of all leaves from R10 and three representative transgenic lines (OE-y1, OE-y2, OE-y3). (D–G) The flowering stage of R10 and *RUBY*-overexpressed plants. (H) The silique and seeds of R10 and OE-y3. (I) The T1 progeny of *RUBY* transgenic lines. (J–K) Quantitative analysis of *RUBY* expression level in leaves and petals. (L–M) Quantification of betalain in leaves and petals. Different letters indicate statistically significant differences at *P* < 0.05 by one-way analysis of variance (ANOVA) with Tukey’s multiple-comparisons test. U.D. represents undetectable.

To create colorful rapeseed, the *35S:RUBY* containing the *CYP76AD1*, *DODA*, and *GT* genes was transferred into yellow-flowered *B. napus* variety R10 by Agrobacterium-mediated hypocotyl transformation. As expected, betalain was successfully synthesized in the callus, seedlings, leaves, flowers, siliques, and seeds (Fig. 1A–H). The phenotype observed in T0 regenerated plants was inherited by their offspring (Fig. 1I). Accordingly, we selected three representative lines and detected the *RUBY* expression levels in leaves and petals, respectively (Fig. 1J and K). The OE-y1 line, which had the lowest expression of *RUBY*, only showed slight red in leaf veins and orange-striped flowers. In line OE-y2 with intermediate expression, the lower leaves were red and the upper leaves were green, with light orange petals. The line with the highest *RUBY* expression, OE-y3, exhibited dark red leaves and dark orange petals (Fig. 1E–G). At the same time, the content of betalain in the leaves and petals was measured, which was highly consistent with *RUBY* expression levels (Fig. 1L and M). These results indicate that varying expression levels of *RUBY* are responsible for different colors. We also detected the genetic and phenotypic stability spanning from T1 to T3 generations. Using droplet digital PCR, we found that OE-y1, OE-y2, and OE-y3 contained multiple, three, and two T-DNA number, respectively, in the T1 generation that showed segregation, and their copy numbers became more stable in higher generations (Supplementary Table 1). The expression level and the betalain contents decreased from T1 to T3 in OE-y1 line (Supplementary Fig. 1A–D), which may be due to the epigenetic regulation in multiple copies transgenic plants. However, the expression and betalain contents kept stable after multiple generations of propagation in OE-y2 and OE-y3 (Supplementary Fig. 1A, C, E, and  F).

### Combination of betalain with carotenoid or chlorophyll produces derivative colors

**Figure 2:**
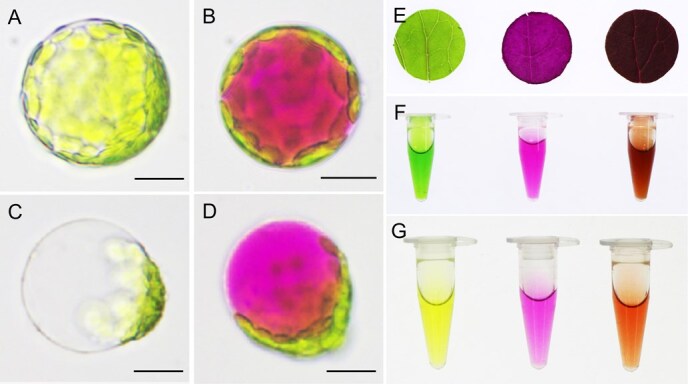
Co-coloration of betalain with carotenoid or chlorophyll. (A and B) The protoplasts from R10 (A) and OE-y3 (B) leaves. (C and D) The vacuole from R10 (C) and OE-y3 (D) ruptured protoplasts. (E) From left to right: betalain removed, chlorophyll removed and untreated leaf disc of OE-y3. (F) Chlorophyll and betalain extracted from OE-y3 leaves and the mixture of chlorophyll and betalain. (G) Carotenoid and betalain extracted from OE-y3 petals and the mixture of carotenoid and betalain. Scale bar = 20 μm.

Interestingly, the leaves and petals in *35S*:*RUBY* overexpression lines did not exhibit the pure betalain red. Instead, the leaves displayed a surprising purplish-black, while the petals were orange (Fig. 1G). Therefore, we supposed that this phenotype may be caused by the synergistic interaction of betalain with background pigments. To validate this hypothesis, we first detected the sub-cellular location of pigments in leaf protoplasts. The red betalain and green chlorophyll can be clearly observed in vacuole and chloroplasts (Fig. 2A–D). Subsequently, we deprived the betalain and chlorophyll from the purplish-black leaves of OE-y3 and obtained green and red leaves as expected (Fig. 2E). In addition, we extracted the green chlorophyll or red betalain using ethanol and water from the transgenic leaves of OE-y3, respectively, and reconstructed the purplish-black by mixing these two pigments *in vitro*, further verifying the mixture effect of green and red (Fig. 2F). Using the same method, we successfully extracted yellow carotenoid and red betalain from orange flowers, and obtained orange color by mixing these two different pigments (Fig. 2G). Taken together, the purplish-black leaves and orange petals are the result of interaction between betalain and chlorophyll or carotenoid, which is highly analogous to the mixture of different pigments during painting.

### Creating true red flowers in *B. napus*

**Figure 3:**
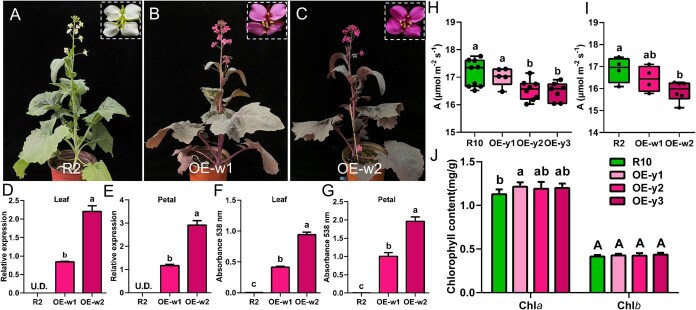
*35S:RUBY* expressed in R2 background. (A–C) The flowering stage of R10 and *RUBY*-overexpressed lines (OE-w1, OE-w2). (D and E) Expression analysis of *RUBY* in leaves and petals. (F and G) The content of betalain in leaves and petals. (H-I) Net photosynthetic rate of *35S:RUBY* transgenic lines in R10 and R2. (J) Chlorophyll *a* and chlorophyll *b* content in R10 transgenic leaves. Different letters indicate statistically significant differences at *P* < 0.05 by one-way analysis of variance (ANOVA) with Tukey’s multiple-comparisons test. U.D. represents undetectable.

To further create red but not orange flowers, we transformed *35S:RUBY* into a white-flowered cultivar R2. We successfully obtained two overexpression lines OE-w1 and OE-w2 (Fig. 3A–C). The expression of *RUBY* in OE-w1 is lower than that in OE-w2 (Fig. 3D and E). As expected, OE-w1 showed lighter leave and flower color than OE-w2 due to less betalain content, consistent with the *RUBY* expression level (Fig. 3F and G). It has been reported that the accumulation of anthocyanin in purple mustard leaves impairs photosynthesis [27]. Therefore, we supposed that the photosynthesis may also be inhibited in betalain-excessively accumulated leaves. As expected, the net photosynthetic rate was found to significantly reduce in *RUBY* high expression lines (OE-y2, OE-y3, and OE-w2) compared with their wild types. While the net photosynthetic rate was comparable with wild type in *RUBY* mild expression lines (OE-y1 and OE-w1) (Fig. 3H and I). The chlorophyll *a* was slightly higher in R10 transgenic lines compared with wild type, and there was no significant difference in chlorophyll *b* (Fig. 3J). The increased chlorophyll *a* might be due to the feedback of the decreased photosynthesis. Thus, we have created rapeseed with strong red flowers but also with inhibited photosynthesis.

### Flower color precise regulation using petal-specific promoter

**Figure 4:**
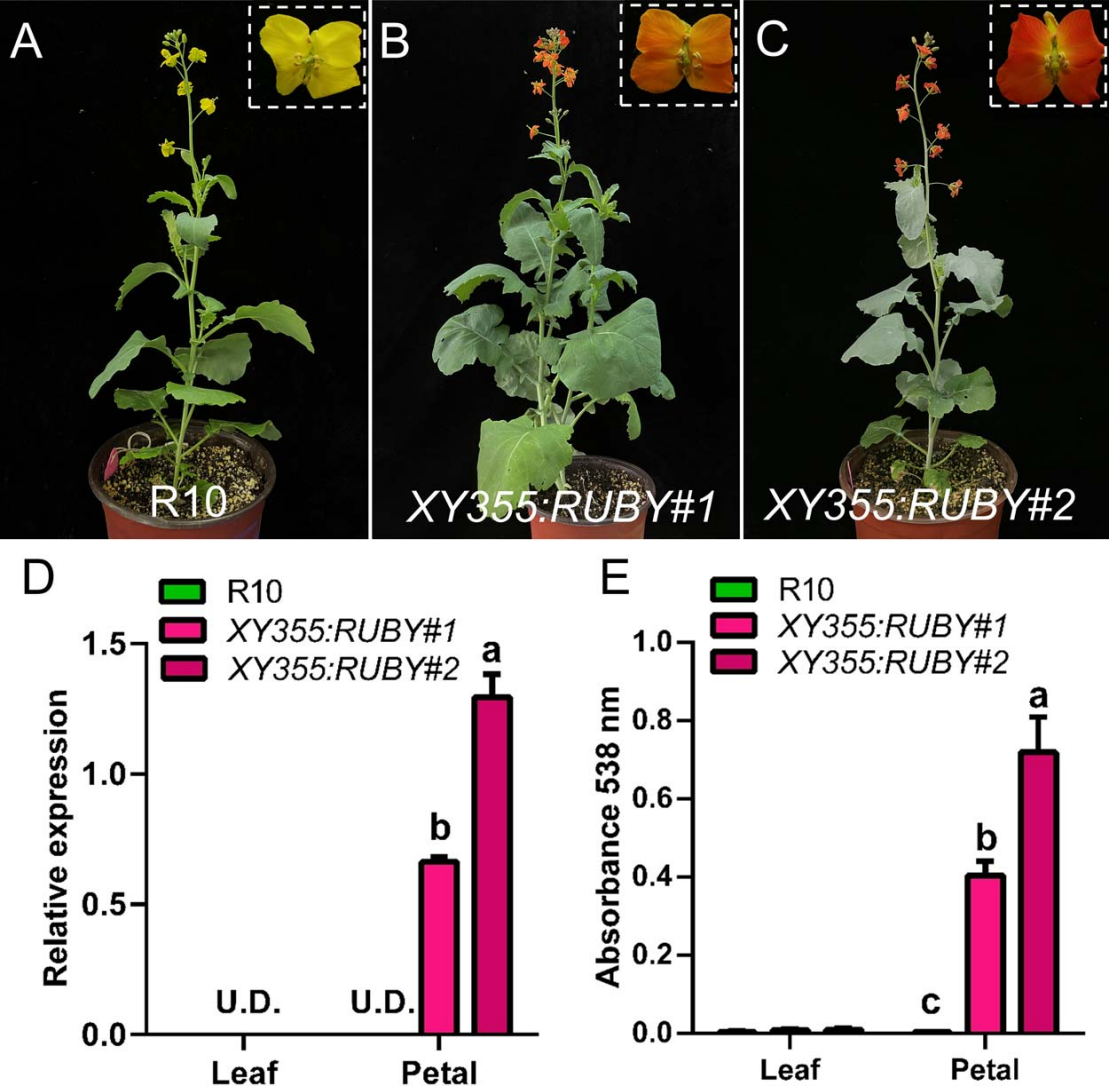
Precise regulation of *RUBY* using the petal-specific promoter *XY355*. (A–C) The flowering stage of R10 and *XY355:RUBY* transgenic lines (*XY355:RUBY#1*, *XY355:RUBY#2*). (D) Expression analysis of *RUBY* in transgenic leaves and petals. (E) The content of betalain in transgenic leaves and petals. Different letters indicate statistically significant differences at *P* < 0.05 by one-way analysis of variance (ANOVA) with Tukey’s multiple-comparisons test. U.D. represents undetectable.

In order to restore the photosynthesis in *RUBY* transgenic plants and enhance the color contrast between petals and leaves, we constructed a new *RUBY* expression vector driven by the petal-specific promoter *XY355*. The *XY355:RUBY* was transformed into the yellow-flowered cultivar R10 and generated two transgenic lines (*XY355:RUBY#1* and *XY355:RUBY#2*). As expected, the transgenic plants showed green leaves with orange flowers (Fig. 4A–C). *RUBY* was specifically expressed in petals, while barely detected in leaves. The line *XY355:RUBY#2* showed higher *RUBY* expression level and more betalain compared with *XY355:RUBY#1* (Fig. 4D and E). These results suggest that using petal-specific promoter can precisely regulate betalain biosynthesis in flowers.

**Figure 5:**
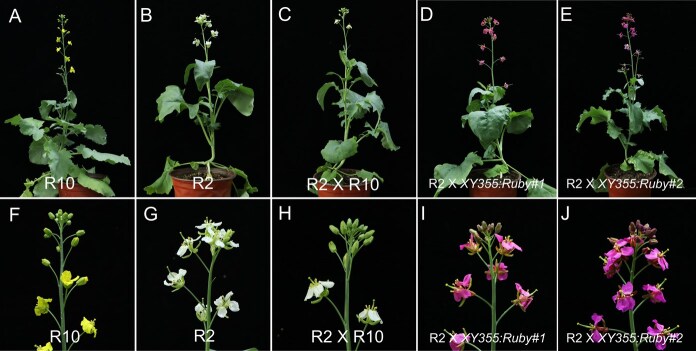
Flower color design through hybridization. (A) Cultivar R10. (B) Cultivar R2. (C) F1 of the cross between R10 and R2. (D and E) F1 of crosses between R2 and *XY355:RUBY#1*, *XY355:RUBY#2*, respectively. (F–J) The inflorescence correspond to the image above.

To create new flower colors with green leaves, we conducted crosses between R2 (white flower) and R10 (yellow flower) firstly. The F1 of R2 × R10 displayed white flowers, demonstrating that white is dominant (Fig. 5A, C, F, and  H). Then, we crossed R2 (female parent) with the orange flower line *XY355:RUBY#1* and *XY355:RUBY#2*, respectively. Due to the dominant effect of white over yellow, the positive F1 had no more yellow carotenoid and showed red petals (Fig. 5D, E, I, and J).

## Discussion

### Three major pigments contribute to the diversity of flower colors

In the plant kingdom, colorful flowers provide visual signals against the plant background to attract different types of insects for pollination, thereby maintaining reproduction and ecological stability [28]. The yellow, red, and purple flowers are attractive to bees and butterflies during the day, while the white are attractive to moths and flies at night [29]. Rapeseed is one of the most important oil crops, with a global harvested area of 42.9 million hectares according to the USDA’s 2024 agricultural statistics. Owing to its profuse flowers and long flowering period, rapeseed has also gradually developed into an important ornamental crop. However, the monochromatic yellow phenotype of most rapeseed flowers underscores the urgency of breeding novel flower color variants, which are essential to satisfy the curiosity and aesthetic preference of different tourists, thus boosting the integrated development of agriculture and tourism.

In contrast to existing colored varieties developed via distant hybridization for anthocyanin pathway introgression, this study introduces betalain biosynthesis through genetic engineering, thereby establishing a novel and versatile approach for creating new ornamental flower colors. Moreover, betalain exhibits lower sensitivity to temperature and pH fluctuations, conferring greater stability and durability to flower colors compared with anthocyanins (Supplementary Fig. 2). Additionally, betalain biosynthesis pathway introduced via genetic engineering is independent of genetic background and free from linkage drag associated with distant hybridization, thus offering distinct advantages in maintaining crop quality and yield. However, the abundant anthocyanin metabolic pathways in existing colored flower varieties provide an important material basis for breeding derivative flower colors with betalain.

**Figure 6:**
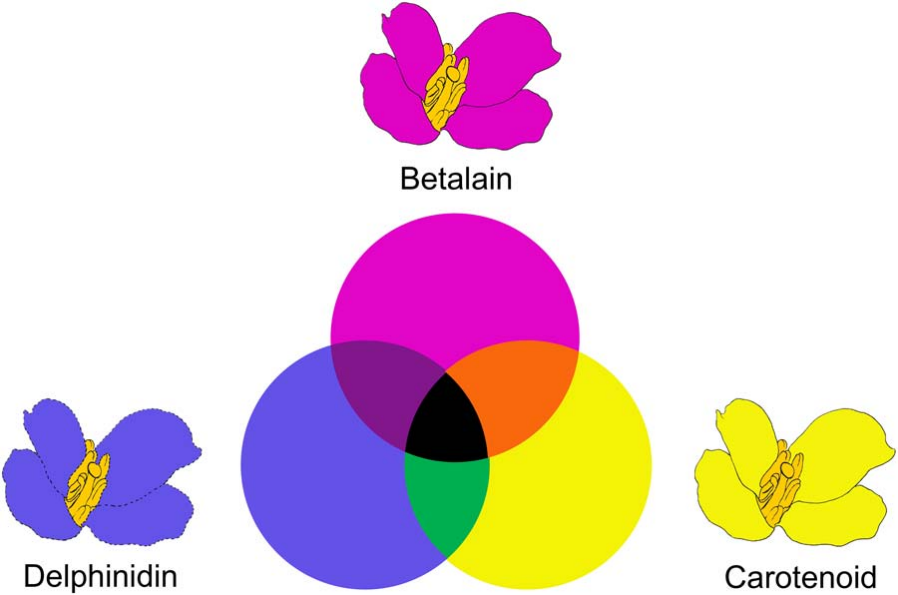
The model of flower color design. Betalain, carotenoid, and delphinidin represent the three major pigments. A series of novel flower colors can be created through introducing different pigments in petals.

As the three major pigment classes, betalain, carotenoid, and anthocyanin collectively regulate plant coloration and the evolutionary diversity of floral colors. *RUBY* has been proved as a powerful tool for effective betalain synthesis in numerous organisms, including rice, Arabidopsis, maize, tomato, and cotton [17, 30, 31]. In this study, when betalain was introduced into white-flowered rapeseed, the flower became red. However, when betalain was introduced into carotenoid abundant yellow-flowered rapeseed, the color turned into orange. In addition to betalain and carotenoid, anthocyanin is another category of pigments for a wide range of colors according to the types, among which cyanidins, pelargonidins, and peonidins are responsible for pink, red, red-purple, whereas delphinidin, petunidin, and malvidin derivatives contribute to the blue and purple colors. The presence of hydroxyl groups on the B-ring of anthocyanidins plays a crucial role in determining the color, with a greater number of hydroxyl groups leading to a shift towards bluer color [32]. Pigment diversity and copigmentation further enhance the richness of colors. For example, mixture of anthocyanins and flavones can result in the shift of longer maximum visible absorption, which make the flower look bluer [33]. We proposed a promising scheme to design derived colors by introducing the three major pigments (Fig. 6). In this model, orange, green, purple, and black can be produced by different pigments combinations, and the main task for the next step is to introduce blue pigments into rapeseed.

### It is promising to create blue flower in rapeseed

Blue is a rare color, among the 10 437 plant species available in the international plant trait database, a total of 772 species (7%) are categorized as “blue” flowers [34]. There are still some natural blue related pigments, such as phycocyanin and delphinidin. However, they are extremely unstable and are easily affected by environmental factors [35]. *F3′5′H*, which encodes an enzyme belonging to CYP75A family, determines hydroxylation patterns of flavanones and regulates the metabolic flux toward delphinidin biosynthesis. This enzyme is highly responsible for blue-hued flowers [32]. In some of the popular cut flower varieties, such as roses, chrysanthemums, carnations, and lilies, *F3′5′H* is absent. Thus, using genetic engineering approach to rescue this gene for the production of blue flowers has drawn significant attention. Holton et al. first isolated *F3′5′H* from petunia flowers [36]. Heterologous expression of the *F3′5′H* in carnations promotes the accumulation of delphinidin-type anthocyanin. In combination with the co-pigmentation with flavone derivatives and a relatively high vacuolar pH of 5.5, the flower color of *F3′5′H* transgenic plants shifts toward blue [22]. Through comparative analysis of expression efficiencies among different combinations of promoters, enhancers, and *F3′5′H* gene variants from diverse sources, significant variations in delphinidin content (0.6%–95.1% of total anthocyanins) were observed in transgenic chrysanthemums, with the optimized combination making the flower color transform from red-purple to purple-violet [24]. Subsequently, the true blue flowers were created by inducing butterfly pea (*Clitoria ternatea*) anthocyanin *3′,5′*-glucosyltransferase (*CtA3′5′GT*), in addition to the expression of *F3′5′H* in chrysanthemums [37].

A notable success in expanding rapeseed floral color diversity is the development of reddish pink/purple-flowered cultivars via interspecific hybridization [38]. It is worth mentioning that the metabolic pathways of flavonoids and anthocyanins are intact in these newly developed colorful rapeseed [26, 39]. In *B. napus* genome, *F3′5′H* is absent, which is directly responsible for the lack of delphinidin-based anthocyanin. These findings of the aforementioned cases provide a promising approach to develop blue flowers by heterologous expression of *F3′5′H* gene together with genes involved in delphinidin modification in current anthocyanin-abundant varieties.

### Enhancing color diversity: precise pigments synthesis

The Cauliflower Mosaic Virus *35S*, actin, and ubiquitin promoters are the most frequently employed constitutive promoters. However, these promoters exhibit strong activity in most tissues, usually resulting in pleiotropic effects on plant growth and development [40]. In this study, *35S:RUBY* transgenic plants displayed a reduction in net photosynthetic rate combined with accumulation of excessive betalain in leaves. Chlorophyll content analysis showed that chlorophyll *a* in the transgenic plants trended to increase. Studies have shown that plants grown under light-shading treatment or weak light conditions will synthesize more chlorophyll as a compensatory response [41]. We hypothesize that the excessive accumulation of betalain disrupts chlorophyll-dependent light harvesting by imposing a shading-like effect, thereby compromising the photosynthetic capacity of leaves. Furthermore, we verified that betalain significantly reduces the leaf transmittance by detecting the light intensity above and below the leaves (Supplementary Fig. 3). Therefore, utilizing inducible or tissue-specific promoters is a more optimal strategy. To date, a substantial number of flower-specific promoters have been identified, such as *XY355* from *B. napus*, *InMYB1* from Japanese morning glory [6, 42]. To recover photosynthesis, we introduced *XY355* promoter and achieved precise betalain biosynthesis in flowers.

The differential distribution of pigments in plant petals leads to the formation of spots or stripes, which further enhances their ornamental value. PsMYB12-bHLH-WD40 complex regulates *PsCHS* regional expression, leading to the formation of colored blotches in tree peony [43]. The formation of three distinct petal spot patterns in *Gorteria diffusa* is achieved through the evolution and expression divergence of three R2R3-MYB transcription factors [44]*. NiorMYB113-1* and *NiorMYB113-2* are restricted to different petal regions and have opposite function in anthocyanin production, resulting in intricate patterns on *Nigella orientalis* petals [45]. In summary, pigment deposition on petals is finely regulated. Precise pigment synthesis not only enables the creation of desired colors but also the generation of color patterns, thereby offering boundless possibilities for breeding novel ornamental flower varieties in the future.

## Materials and methods

### Plant materials and culture conditions

A white-flowered cultivar R2 and a yellow-flowered cultivar R10 were used as transgenic receptors. R2 and R10 are short-life period verities, no need vernalization. ZS11 cultivar was used for *XY355* promoter amplification. All the transgenic plants were planted in a growth chamber at 25°C with16h light/8 h dark under normal planting management.

### Vector construction and *B. napus* transformation

The *35S:RUBY* vector was described previously [17]. To construct *XY355:RUBY* expression cassette*,* the petal-specific promoter *XY355* (318 bp) was amplified from *B. napus* ZS11 cultivar genome DNA using primers XY355RubyF and XY355RubyR (Supplementary Table 2), and then replaced *35S* promoter through seamless cloning. *35S:RUBY* was transformed into R2 and R10, and *XY355:RUBY* was transformed into R10 by *Agrobacterium*-mediated genetic transformation. The positive transgenic plants of *XY355:RUBY* were screened out using promoter specific forward primer XY355RubyF and the *RUBY* specific primer 35SRubyCX3 (Supplementary Table 2).

### RNA isolation and quantitative RT-PCR

Young leaf and petal RNA was extracted using plant RNA Kit (Omega, R6827-02). And then, RNA was reverse transcribed into cDNA using PrimeScript RT reagent Kit (TaKaRa, RR047A), referring to the manufacturer’s user manual. qRT-PCR was conducted using SYBR Green Master Mix (Vazyme, Q712) with a LightCycler 96 Real-Time PCR detection system (Roche). *BnaActin* was used as internal control. Three biological replicates were conducted for each sample. The primers for qRT-PCR analysis are listed in Supplementary Table 1.

### Betalain extraction and quantification analysis

For betalain extraction, fresh leaves or petals were frozen and ground into powder in liquid nitrogen. During extraction, 100 mg powder of each sample was completely immersed into 2 ml 95% ethanol for 30 min and removed the supernatant by centrifugation (12 000 g, 5 min), repeated once until all chlorophyll or carotenoid was removed. And then the remaining precipitate was resuspended in 2 ml distilled water and vortexed well, and then centrifuged at 12 000 g for 5 min subsequently. The supernatant was used for betalain analysis by measuring the absorbance value at 538 nm wavelength using a micro spectrophotometer (Biotek Epoch).

### Isolation of rapeseed protoplasts

To isolate protoplasts, the fully expanded leaves from three-week-old seedlings were used. The formulation for preparing 10 mL enzyme solution is as follows: 0.15 g cellulase R10 (Yakult), 0.04 g macerozyme R10 (Yakult), 4 ml 1 mol/L mannitol, 1 mL 0.2 mol/L KCl, 2 ml 0.1 mol/L MES (pH, 5.7), 200 μl 0.5 mol/L CaCl_2_, 500 μL 2% BSA, and sterile water was added to a final volume of 10 ml. The leaves were cut into thin strips and immersed in enzyme solution, then the mixture was vacuum-infiltrated for 30 min followed by 2-h digestion in the dark. The protoplasts were filtered through cheesecloth and washed with W5 solution (154 mmol/L NaCl, 125 mmol/L CaCl_2_, 5 mmol/L KCl and 2 mmol/L MES (pH 5.7) and 5 mmol/L glucose). Finally, the protoplasts were observed and imaged using a microscope (LEICA EX30).

### Determination of net photosynthetic rate and chlorophyll content

The net photosynthetic rate of leaves was measured using an LI-COR portable photosynthesis measurement system (LI-6800, USA). The parameters were set as follows: flow rate 700 μmol s^−1^, CO_2_ concentration 400 μmol mol^−1^, light intensity 1200 μmol m^−2^ s^−1^, detection chamber temperature 25°C, and humidity 70%. The fully expanded leaves were used for measurement from 9:00 a.m. to 11:00 a.m. Chlorophyll detection was performed according to previous report [46]. Briefly, the leaves were ground into powder, and approximately 100 mg of the sample was weighed and extracted with 95% ethanol on ice. The content of chlorophyll *a* and chlorophyll *b* was calculated according to the absorbance values at 665 and 649 nm.

### Removal of chlorophyll and betalain in leaves

Leaf discs were obtained by using a hole punch on the same leaf. To remove the betalain, the leaf discs were first immersed in a water bath at 95°C for 30 s. Then, the leaf discs were repeatedly pressed under flow tap water to remove all the betalain. To remove the chlorophyll, the circular leaf discs were soaked in absolute ethanol and immersed in a water bath at 60°C for 30 min with shaking to remove the chlorophyll.

### Droplet digital PCR

Droplet digital PCR (ddPCR) was performed as described previously [47]. *HPT* (hygromycin) gene was used as the marker gene, and *CruA* gene was used as the reference gene. The 20 μl reaction system for ddPCR consists of: 10 μl 2× ddPCR Supermix (Bio-Rad), 2 μl primer/probe mixture, 2 μl genome DNA (10–20 ng/μl) and 6 μl distilled water. The 20 μl reaction system and 70 μl droplet generation oil (Bio-Rad) are used to generate water-in-oil emulsions via the droplet generator (Bio-Rad, QX200). The generated droplets were transferred to a 96-well plate and amplified using PCR thermal cycler (Bio-Rad, C1000) with the following program: initial denaturation at 95°C for 10 min; followed by 40 cycles of denaturation at 94°C for 30 s and annealing/extension at 57°C for 1 min. After cycling, the PCR plate was incubated at 98°C for 10 min and then cooled to 4°C. Afterwards, the 96-well plate was placed in Droplet Reader (Bio-Rad, QX200) to count the number of positive and negative droplets. The transgenic copy number can be determined by calculating the ratio of the marker gene to the reference gene.

## Supplementary Material

Web_Material_uhaf193

## Data Availability

Supplementary information for this article can be found online.
