# Supplementary material for: Precise pigment biosynthesis for flower color design in Brassica napus
Source: Hortic Res. 2025 Jul 29;12(10):uhaf193. doi: 10.1093/hr/uhaf193 (PMC12541713; doi:10.1093/hr/uhaf193)
Supplement: Web_Material_uhaf193 [file web_material_uhaf193.zip › Supplemental Figure.docx]

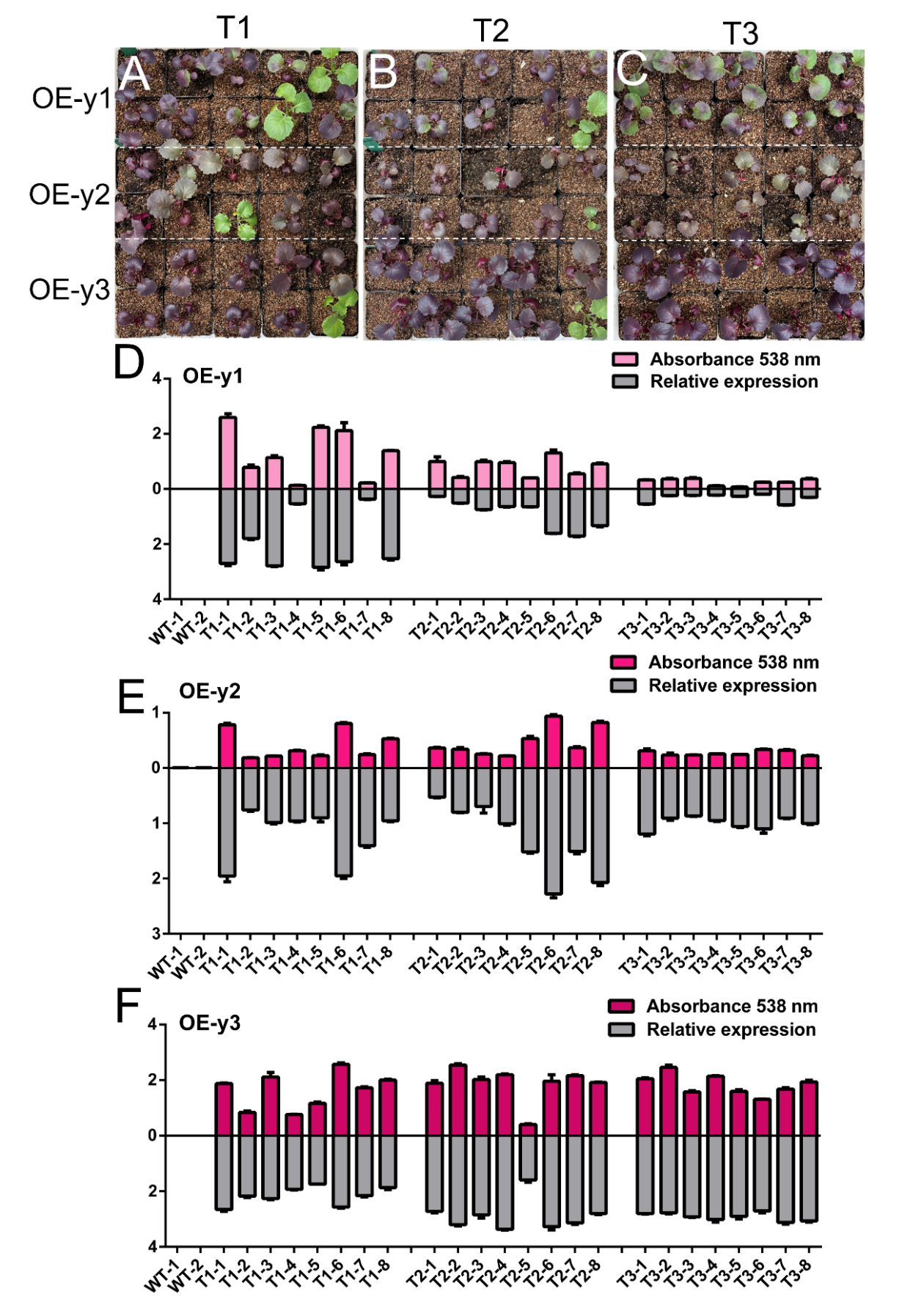
**Supplemental Figure 1.** The genetic and phenotypic stability assay spanning from T1 to T3 generations.

(A-C) The seedling phenotypes of OE-y1/y2/y3 from T1 to T3 generations. (D-F) Analysis of *RUBY* expression levels and betalain content in OE-y1/y2/y3 from T1 to T3 generations. For each line, 8 individuals were used and the bar represents the standard error of three samples from the same individual.


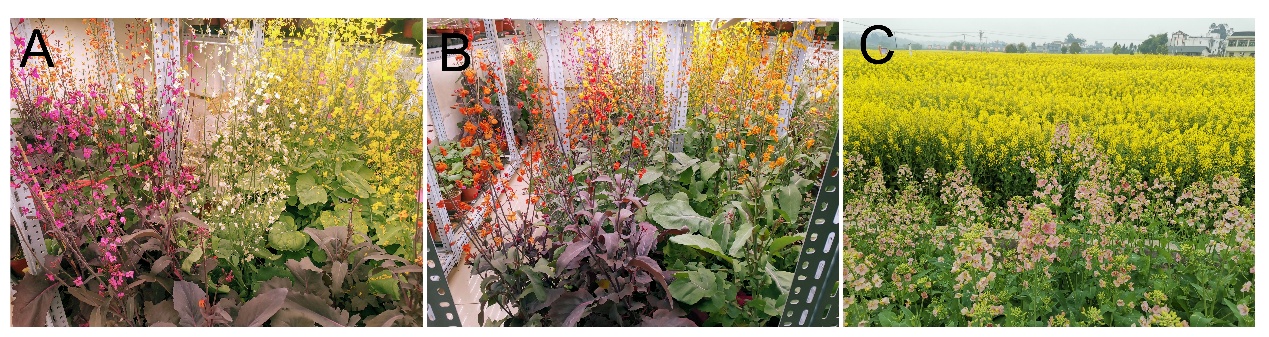


**Supplemental Figure 2.** The phenotype of *35S:RUBY* overexpression plants and commercial colorful flower cultivars at the flowering stage.

(A) The *35S:RUBY* overexpression plants in white flowered R2. (B) The *35S:RUBY* overexpression plants in yellow flowered R10. (C) The commercial colorful flower cultivars.


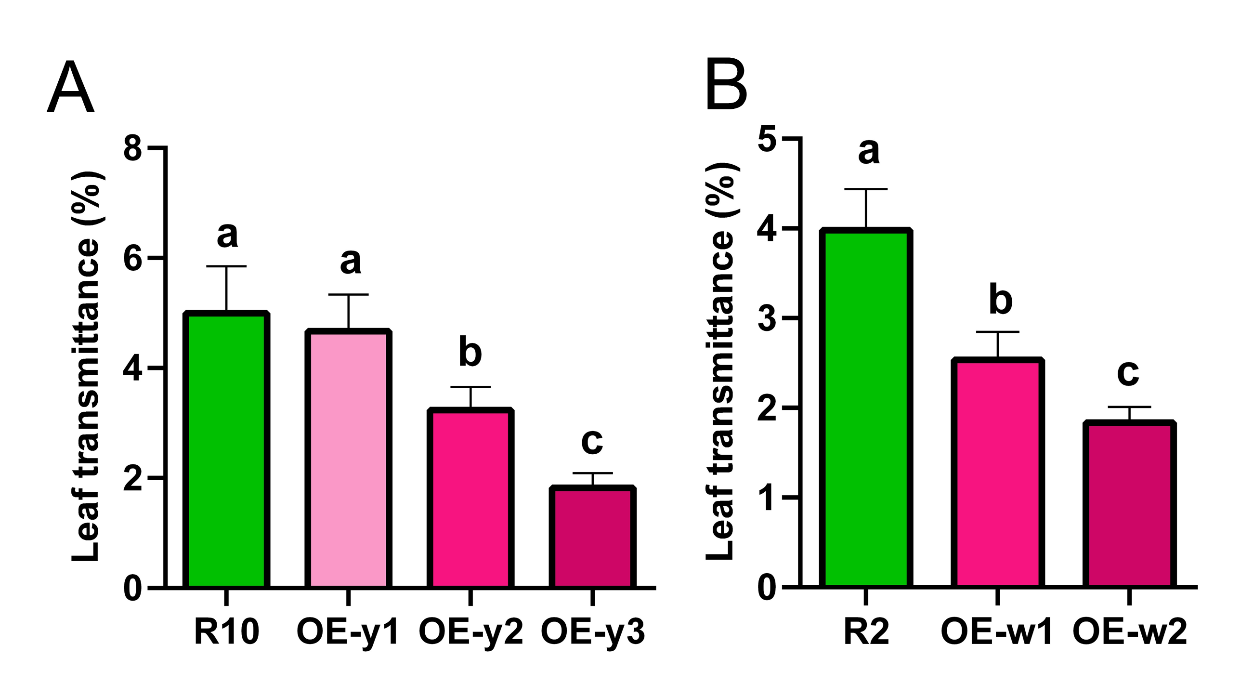


**Supplemental Figure 3.** The leaf transmittance analysis between WT and *35S:RUBY* overexpression lines.

(A) The leaf transmittance in R10 transgenic leaves. (B) The leaf transmittance in R2 transgenic leaves. Different letters indicate statistically signiﬁcant differences at *P* < 0.05 by one-way analysis of variance (ANOVA) with Tukey’s multiple-comparisons test. The leaf transmittance was calculated as the ratio of light intensity below leaves to that above leaves.
