# Supplementary material for: Precise pigment biosynthesis for flower color design in Brassica napus
Source: Hortic Res. 2025 Jul 29;12(10):uhaf193. doi: 10.1093/hr/uhaf193 (PMC12541713; doi:10.1093/hr/uhaf193)
Supplement: Web_Material_uhaf193 [file web_material_uhaf193.zip › Supplemental Table.docx]

**Supplemental Table 1. The T-DNA number determination in transgenic plants by ddPCR**

| **Lines** | **Generations** | | **Individuals** | **Gene** | **Droplet detection** | | | **Poisson statistics** | | | **Ratio** | **T-DNA number** |
| --- | --- | --- | --- | --- | --- | --- | --- | --- | --- | --- | --- | --- |
|  |  |  |  |  | **Accepted** | **Positives** | **Negatives** | **Maximum** | **Minimum** | **Copies** |  |  |
| **OE-y1** | **T1** | **1** | | ***HPT*** | **16330** | **8115** | **8215** | **826** | **790** | **808** | **1.76** | **7** |
|  |  |  |  | ***CruA*** | **19385** | **6247** | **13138** | **469** | **452** | **458** |  |  |
|  |  | **2** | | ***HPT*** | **16262** | **15630** | **632** | **3910** | **3730** | **3820** | **3.82** | **15** |
|  |  |  |  | ***CruA*** | **18946** | **10851** | **8095** | **1020** | **991** | **1000** |  |  |
|  |  | **3** | | ***HPT*** | **15507** | **15044** | **463** | **4240** | **4030** | **4130** | **5.71** | **23** |
|  |  |  |  | ***CruA*** | **19372** | **8898** | **10474** | **739** | **716** | **723** |  |  |
|  | **T2** | **1** | | ***HPT*** | **19244** | **17651** | **1593** | **2990** | **2880** | **2930** | **2.62** | **10** |
|  |  |  |  | ***CruA*** | **21555** | **13233** | **8322** | **1140** | **1110** | **1120** |  |  |
|  |  | **2** | | ***HPT*** | **19539** | **17542** | **1997** | **2733** | **2635** | **2683** | **4.27** | **17** |
|  |  |  |  | ***CruA*** | **20670** | **8553** | **12117** | **642** | **621** | **628** |  |  |
|  |  | **3** | | ***HPT*** | **13688** | **12974** | **714** | **3560** | **3390** | **3470** | **5.83** | **23** |
|  |  |  |  | ***CruA*** | **19335** | **7671** | **11664** | **608** | **588** | **595** |  |  |
|  | **T3** | **1** | | ***HPT*** | **16277** | **14822** | **1455** | **2910** | **2790** | **2845** | **6.82** | **27** |
|  |  |  |  | ***CruA*** | **19177** | **5725** | **13452** | **428** | **411** | **417** |  |  |
|  |  | **2** | | ***HPT*** | **19029** | **18513** | **516** | **4350** | **4150** | **4240** | **7.21** | **29** |
|  |  |  |  | ***CruA*** | **19840** | **7806** | **12034** | **601** | **581** | **588** |  |  |
|  |  | **3** | | ***HPT*** | **19740** | **18851** | **889** | **3730** | **3570** | **3650** | **8.24** | **33** |
|  |  |  |  | ***CruA*** | **20783** | **6521** | **14262** | **454** | **437** | **443** |  |  |
| **OE-y2** | **T1** | **1** | | ***HPT*** | **19632** | **6146** | **13486** | **453** | **431** | **442** | **0.52** | **2** |
|  |  |  |  | ***CruA*** | **20865** | **10668** | **10197** | **859** | **834** | **842** |  |  |
|  |  | **2** | | ***HPT*** | **19372** | **14854** | **4518** | **1743** | **1683** | **1713** | **1.04** | **4** |
|  |  |  |  | ***CruA*** | **19835** | **14959** | **4876** | **1680** | **1636** | **1651** |  |  |
|  |  | **3** | | ***HPT*** | **15363** | **9216** | **6147** | **1101** | **1055** | **1078** | **1.20** | **5** |
|  |  |  |  | ***CruA*** | **21358** | **11378** | **9980** | **912** | **887** | **895** |  |  |
|  | **T2** | **1** | | ***HPT*** | **15435** | **8052** | **7383** | **887** | **848** | **868** | **1.00** | **4** |
|  |  |  |  | ***CruA*** | **19572** | **10152** | **9420** | **878** | **852** | **860** |  |  |
|  |  | **2** | | ***HPT*** | **15580** | **10051** | **5529** | **1244** | **1194** | **1219** | **1.27** | **5** |
|  |  |  |  | ***CruA*** | **19820** | **11034** | **8786** | **976** | **948** | **957** |  |  |
|  |  | **3** | | ***HPT*** | **19054** | **15511** | **3543** | **2015** | **1945** | **1979** | **1.50** | **6** |
|  |  |  |  | ***CruA*** | **20726** | **13963** | **6763** | **1341** | **1306** | **1318** |  |  |
|  | **T3** | **1** | | ***HPT*** | **17621** | **9945** | **7676** | **998** | **958** | **978** | **1.47** | **6** |
|  |  |  |  | ***CruA*** | **18055** | **7785** | **10270** | **679** | **656** | **664** |  |  |
|  |  | **2** | | ***HPT*** | **19662** | **15687** | **3975** | **1914** | **1849** | **1881** | **1.47** | **6** |
|  |  |  |  | ***CruA*** | **19781** | **13099** | **6682** | **1300** | **1265** | **1277** |  |  |
|  |  | **3** | | ***HPT*** | **13414** | **9819** | **3595** | **1582** | **1517** | **1549** | **1.55** | **6** |
|  |  |  |  | ***CruA*** | **19588** | **11204** | **8384** | **1018** | **989** | **998** |  |  |
| **OE-y3** | **T1** | **1** | | ***HPT*** | **17321** | **3312** | **14009** | **258** | **241** | **250** | **0.26** | **1** |
|  |  |  |  | ***CruA*** | **21268** | **11847** | **9421** | **976** | **949** | **958** |  |  |
|  |  | **2** | | ***HPT*** | **17478** | **7251** | **10227** | **645** | **616** | **630** | **0.47** | **2** |
|  |  |  |  | ***CruA*** | **20770** | **14182** | **6588** | **1375** | **1339** | **1351** |  |  |
|  |  | **3** | | ***HPT*** | **17707** | **8696** | **9011** | **812** | **778** | **795** | **1.00** | **4** |
|  |  |  |  | ***CruA*** | **19470** | **9530** | **9940** | **807** | **783** | **791** |  |  |
|  | **T2** | **1** | | ***HPT*** | **19645** | **6773** | **12872** | **509** | **486** | **497** | **0.52** | **2** |
|  |  |  |  | ***CruA*** | **19949** | **11088** | **8861** | **973** | **945** | **955** |  |  |
|  |  | **2** | | ***HPT*** | **8530** | **2159** | **6371** | **358** | **336** | **343** | **0.75** | **3** |
|  |  |  |  | ***CruA*** | **18633** | **6010** | **12623** | **470** | **447** | **458** |  |  |
|  |  | **3** | | ***HPT*** | **18302** | **6328** | **11974** | **512** | **487** | **499** | **0.98** | **4** |
|  |  |  |  | ***CruA*** | **18930** | **6645** | **12285** | **521** | **496** | **509** |  |  |
|  | **T3** | **1** | | ***HPT*** | **17027** | **6831** | **10196** | **618** | **589** | **603** | **1.07** | **4** |
|  |  |  |  | ***CruA*** | **19701** | **7500** | **12201** | **577** | **557** | **564** |  |  |
|  |  | **2** | | ***HPT*** | **17383** | **6071** | **11312** | **518** | **493** | **505** | **1.03** | **4** |
|  |  |  |  | ***CruA*** | **20307** | **6919** | **13388** | **502** | **484** | **490** |  |  |
|  |  | **3** | | ***HPT*** | **17460** | **12028** | **5432** | **1400** | **1348** | **1374** | **0.98** | **4** |
|  |  |  |  | ***CruA*** | **18581** | **12909** | **5672** | **1422** | **1383** | **1396** |  |  |

Note: In this detection system, the number of reference gene *CruA* (AnAnCnCn) is 4, the T-DNA number of single copy heterozygote (Aa genotype) is recorded as 1, the homozygote (AA genotype) is recorded as 2.

| Primer name | Sequence (5’-3’) | Annotation |
| --- | --- | --- |
| XY355RubyF | AAACACTGATAGTTTACTAGTGTCGACTAAATTGAAACAGAAAG | *XY355:RUBY* construction |
| XY355RubyR | GGTCGCATGATCCATACTAGTGAAGGTAGTGTGGTTGTTTGA | *XY355:RUBY* construction |
| 35SRubyCX3 | GAAGCAGCTTGGTGGTCTGCT | positive detection |
| BnaActin-F | GGCTCCTCTTAACCCAAAGGC | qRT-PCR |
| BnaActin-R | CACACCATCACCAGAATCCAGC | qRT-PCR |
| RubyqRT-F | AACAGCATCCTTGAGTCTCTTCG | qRT-PCR |
| RubyqRT-R | TTCTCTTTGGAGATCTCGCCTTC | qRT-PCR |

**Supplemental Table 2. Primers used in this study**
